# Supplementary material for: Deriving Information on Play and Playfulness of 3–5-Year-Olds from Short Written Descriptions: Analyzing the Frequency of Usage of Indicators of Playfulness and Their Associations with Maternal Playfulness
Source: Behav Sci (Basel). 2022 Oct 8;12(10):385. doi: 10.3390/bs12100385 (PMC9598545; doi:10.3390/bs12100385)
Supplement: Supplementary file 1 [file behavsci-12-00385-s001.zip › Information S1 (accordance with playfulness models).pdf]

Information S1

Accordance of the playfulness criteria based on mothers' free descriptions with playfulness models in children and youth

Method

We had the free texts rated by two external student raters to the extent they match with the existing playfulness models know for children and youth. This is the Liebermann model [7; further developed by 2] with the five playfulness dimensions physical, social, and cognitive spontaneity as well as manifest joy and sense of humor (see also section introduction) and the OLIW-model [8,31] with the playfulness dimensions Other-directed, Lighthearted, Intellectual, and Whimsical (see also section introduction). Therefore, we first assigned our playfulness characteristics tentatively to these playfulness models. These assignments are displayed in Table S2.1.

Table S2.1

Tentative assignments of the playfulness characteristics to the existing Playfulness models in children and adolescents.

Playfulness model: Proyer [8; OLIW]; 31 [OLIW-Youth]

OLIW_O	OLIW_L	OLIW_I	OLIW_W
1. actively initiating humor<br>2. playful exchange with others	5. carelessness<br>6. risk-taking<br>7. need for movement<br>8. urge to talk*	3. widespread interests<br>4. active and explorative behavior<br>9. creativity<br>10. imagination<br>11. cognitive spontaneity<br>12. to do things on his/her own in a playful way	13. enjoying to fool around and be silly<br>14. own style<br>15. willpower

Playfulness model: Lieberman [28,35; extended by 2]

Physical Spontaneity	Social Spontaneity	Cognitive Spontaneity	Manifest Joy	Sense of Humor
4. active and explorative behavior<br>7. need for movement<br>6. risk-taking**	2. playfull exchange with others<br>15. willpower	3. widespread interests<br>9. creativity<br>10. imagination<br>11. cognitive spontaneity<br>(12. to do things on his/her own in a playful way***)<br>14. own style	5. carelessness<br>8. urge to talk<br>12. to do things on his/her own in a playful way***	1. actively initiating humor<br>13. enjoying to fool around and be silly

Note. * Due to its social component, this characteristic can alternatively be assigned to other-directed playfulness. ** According to our analysis, this characteristic does not fit very well into this playfulness model. *** Due to the descriptive characteristic of quality, this characteristic can also be assigned to Cognitive Spontaneity.

For the ratings, in each model and for each playfulness dimension, one item was selected to represent this dimension. Items were selected according to selectivity and usability for children descriptions. The external raters had to decide the extent to which the content of this item/dimension was reflected in the mothers' free descriptions. The representative items are displayed in Table S2.2.

Table S2.2

The playfulness items selected for external ratings according to two playfulness models

Playfulness dimension	Selected item
Playfulness model: OLIW [8,31]	
Other-directed	He/she has close friends with whom he/she can just fool around und be silly.
Lighthearted	He/she is a lighthearted person.
Intellectual	If he/she has to learn something new under time pressure, he/she tries to find a playful way to think about the topics—this helps him/her learn.
Whimsical	He/she likes to swim "against the stream."
Playfulness model according to Liebermann [7; extended by 2]	
Physical Spontaneity	The child engages in spontaneous physical movement and activity during play.
Social Spontaneity	While playing, the child shows flexibility in his/her interactions with the surrounding structure.
Cognitive Spontaneity	The child shows spontaneity during expressive and dramatic play.
Manifest Joy	The child does show joy in or during his/her play activities.
Sense of Humor	The child shows a sense of humor during play.

Note. Items of the OLIW-model ranged from 1 ('not at all') to 7 ('totally agree') and of the Liebermann/Barnett-model from 1 ('doesn't sound at all like the child') to 5 ('sounds exactly like the child').

Results

For the OLIW-model, in general, the playfulness characteristics based on mothers' free descriptions are reflected in the dimensions of the OLIW-model. We obtained small to middle associations (Table S2.3), whereby for three out of four dimensions (Other-directed, Lighthearted and Whimsical playfulness), the correlations within the dimensions are numerically higher than the correlations with other dimensions. However, there are significant associations with other dimensions (e.g., Intellectual playfulness).

Table S2.3

Accordance of the obtained playfulness characteristics (dimensions) with the existing playfulness models (based on external ratings)

Playfulness model: Proyer [8; OLIW]; [OLIW-Youth; 31]

Dimensions / External ratings	O	L	I	W
O	$.39^{***}$	$.18^{*}$	$.25^{***}$	.05
L	.09	$.22^{**}$	$.15^{*}$	.07
I	$.22^{**}$	$.26^{***}$	$.24^{***}$	-.05
W	-.02	$.22^{**}$	.10	$.23^{**}$

Playfulness model: Lieberman [28,35; extended by 2]

Dimensions / External ratings	Physical S	Social S.	Cognitive S.	Manifest Joy	Humor
Physical S.	.44***	.01	.02	.07	.01
Social S.	.00	.43***	.14*	.07	.17*
Cognitive S.	.09	.24***	.63***	.37***	.12
Manifest Joy	.27***	.08	.21**	.14*	.24***
Humor	.18*	-.01	.11	.10	.64***

Note. Dimensions = are based on tentative assignments of the playfulness characteristics to the playfulness dimensions of the playfulness models. External ratings = mean ratings of two independent student raters based on 1-item for each playfulness dimension (see Table 4). S.=Spontaneity.

$^{*}p < 0.05$. $^{**}p < 0.01$. $^{***}p < 0.001$.
