# Supplementary material for: Deriving Information on Play and Playfulness of 3–5-Year-Olds from Short Written Descriptions: Analyzing the Frequency of Usage of Indicators of Playfulness and Their Associations with Maternal Playfulness
Source: Behav Sci (Basel). 2022 Oct 8;12(10):385. doi: 10.3390/bs12100385 (PMC9598545; doi:10.3390/bs12100385)
Supplement: Supplementary file 1 [file behavsci-12-00385-s001.zip › Table S1 (mothers playfulness and control variables ).pdf]

Table S1

Distributions and statistics of mothers‘ self-reported playfulness across socioemotional features of children and mothers

		Mothers‘ self-reported playfulness				
		**O**	**L**	**I**	**W**	**SMAP**
Sociodemographic featuers		*M* (*SD*)	*M* (*SD*)	*M* (*SD*)	*M* (*SD*)	*M* (*SD*)
Child's gender						
Female		4.65 (0.90)	4.03 (1.02)	3.59 (1.12)	3.95 (1.05)	3.96 (1.22)
Male		4.56 (1.03)	3.82 (0.92)	3.49 (1.02)	3.81 (0.89)	3.88 (1.24)
	t*(*df*); *p	0.66(206); .51	1.56(206); .12	0.68(206); .50	1.06(206); .29	0.47(206); .64
Child's age						
3		4.58 (1.03)	3.89 (1.02)	3.45 (1.02)	3.81 (1.00)	3.91 (1.25)
4		4.59 (0.93)	3.93 (0.95)	3.65 (1.18)	3.90 (0.97)	3.86 (1.29)
5		4.64 (0.97)	3.90 (0.93)	3.51 (1.01)	3.92 (0.92)	3.98 (1.16)
	F*(*df*); *p	0.06 (2,205); .94	0.03 (2,205); .97	0.65 (2,205); .53	0.27 (2,205); .76	0.15(2, 205); .86
Child's siblings						
0		4.68 (0.81)	3.79 (1.00)	3.59 (0.98)	3.86 (0.89)	3.97 (1.15)
1		4.49 (1.11)	3.85 (0.94)	3.32 (1.07)[a]	3.75 (0.98)	3.85 (1.33)
2		4.60 (1.02)	4.13 (0.96)	3.77 (1.18)	4.00 (0.97)	3.98 (1.21)
3-4		4.97 (0.58)	4.54 (0.59)	4.11 (0.97)	4.60 (1.12)	3.82 (1.31)
	F*(*df*); *p	0.99(3,204); .40	2.47(3,204); .06	**3.05(3,204); .04**	2.43(3,204); .67	0.19(3, 204); .91
Mothers' age						
	R; p	**-.20; .00**	-.03; .66	.01; .87	**-.15; .03**	**-.23; .00**

Mothers' family status						
Single/separated		4.94 (0.70)	4.02 (0.89)	3.71 (0.94)	4.39 (0.73)	4.32 (1.14)
Relationship/married		4.56 (1.00)	3.89 (0.98)	3.51 (1.08)	3.81 (0.97)	3.87 (1.24)
	t(df); p	**2.36(206); .02**	0.60(206); .55	0.86(206); .39	**2.80(206); .01**	1.68(206); .10
Mother's educational level						
No degree/lower school tracks		4.76 (0.91)	4.00 (0.83)	3.36 (1.15)	3.93 (0.92)	4.00 (1.45)
A-levels		4.54 (0.98)	3.63 (0.99)	3.58 (0.98)	3.68 (0.98)	3.90 (1.13)
University degree		4.60 (1.01)	3.90 (1.03)	3.62 (1.12)	3.91 (0.95)	3.85 (1.21)
Vocational training		4.64 (0.94)	4.09 (0.90)	3.50 (0.97)	3.87 (1.00)	4.12 (1.18)
	F(df); p	0.24(3,192); .87	1.42(3,192); .24	0.42(3,192); .74	0.45(3,192); .72	0.56(3, 192); .64
Mothers' time spent with child						
	r, p	.00, .96	.13; .07	-.06; .36	.04; .53	.07; .31

Note. *N* = 208 mothers. [a] Post-hoc tests (LSD) revealed significant lower scores for group 1 siblings compared to the groups of 2 siblings and 3-4 siblings. SMAP (Short Measure of Adult Playfulness) = Global Playfulness. O = Other-directed. L = Lighthearted. I = Intellectual. W = Whimsical. Higher scores indicate higher endorsement.
